# Anterior cerebral falx plane in MR images to estimate the craniofacial midline

**DOI:** 10.1038/s41598-023-42807-6

**Published:** 2023-10-01

**Authors:** Jun Pei, Xu Liao, Lingling Ge, Jianwei Liu, Xiling Jiang

**Affiliations:** Affiliated Hospital of Chifeng University, Yuanlin Road 98, Chi Feng, 150400 Neimenggu China

**Keywords:** Developmental biology, Anatomy

## Abstract

Multiple methods have been proposed for evaluating the symmetry of facial contour by utilizing the median sagittal plane of the skull as a reference and measuring the maxillofacial region. To replace the manual mark point analysis method, we used the anterior cerebral falx plane in MRI images as an indicator of the craniofacial midline. The MRI examination data of 30 individuals were analyzed with a MeVisLab workstation. Two independent examiners performed 15 anthropometric measurements (4 angular, 11 linear) and compared the MRI-based anterior cerebral falx plane with the manual mark point analysis of the craniofacial midline estimation. All measurements were repeated after 3 weeks. Statistical analyses included the repeatability and reproducibility of the 2 methods based on intra-observer and inter-observer correlation coefficients (ICCs), respectively. Precision was estimated by intergroup comparison of the coefficient of variation. The anterior falx plane derived from the MRI data resulted in an intra-observer ICC of 0.869 ± 0.065 (range 0.733–0.936) and inter-observer ICC of 0.876 ± 0.0417 (0.798–0.932) for all measurements, showing significant correlations with the ICC values obtained by the mark point method (p < 0.05). The coefficient of variation showed that the precisions of the 2 methods were statistically comparable. We conclude that, for MRI-based craniofacial midline estimation, measurements made using the anterior cerebral falx plane are as precise, repeatable, and reproducible as those using the manual mark point analysis method. It has a high potential for application in radiation-free 3-dimensional craniofacial analysis.

## Introduction

The aesthetics of the human face greatly depend on the symmetry of facial contour^1,2^. Symmetry is also an important standard for evaluating the outcome of orthognathic and maxillofacial surgery^3^. Three-dimensional (3D) imaging is increasingly used for clinical diagnosis, surgical simulation, and perioperative evaluation of the skull and maxillofacial region, and can be useful for determining facial symmetry. Promising outcomes have been demonstrated using emerging techniques such as cone beam computed tomography (CBCT) and various image post-processing algorithms^4–6^. Methods that are still in the initial stages of research include stereo-photogrammetry, morphanalysis, and laser scanning^7^.

An advantage of 3D-imaging is magnification and reduced distortion^8^. Combined surface and hard tissue information aids facial evaluation^9^. Facial analysis must rely on reproducible landmarks^10^. Quantitative evaluation of 3D craniofacial images requires a reliable midline or median sagittal plane^7^. CBCT has achieved high accuracy in measuring facial symmetry using multiple anatomical landmarks and is highly accurate for measuring facial symmetry^11^, but the radiation exposure of CBCT is significantly higher compared with traditional 2-D radiography (albeit lower than that of computed tomography)^12^. For these reasons there are few available 3D imaging studies for craniofacial analysis^13^. Analytical methods for 3D imaging-based craniofacial analysis are still in the early stage^14–17^. Standardized landmarks for facial symmetry measurements are needed^18^, and a database needs further development.

Many methods have been proposed for assessing the median sagittal plane of the skull and maxillofacial region^19–24^. These can be roughly categorized as manual mark point, ontology-mirror, or computer automation^25^. The first is time-consuming and laborious, whereas the latter 2 have device and software requirements that inhibit their broad application. Magnetic resonance imaging (MRI) may be helpful to estimate the midline. The soft and bone tissue sequences provided by MRI enable observations of nerves and muscles, and hard tissue is displayed on bone sequences. In addition, MRI forgoes the risk of radiation exposure^26^. MR images can depict the forebrain, frontal, and upper middle face in the same signal with a highly consistent midline.

To establish the median sagittal plane for craniofacial analysis, Jiang et al.^27^ proposed using the plane formed by the anterior falx cerebri (i.e., the anterior cerebral falx plane), the sickle-shaped intracranial structure formed by invagination of the dura mater between the cerebral hemispheres. However, the diagnostic performance of this method has not been quantitatively verified and agreement with conventional methods of midline estimation has not been addressed. Accordingly, to provide a theoretical basis for its application in 3D craniofacial analysis, the present study investigated the repeatability and reproducibility of the measurements obtained using the anterior cerebral falx plane, gained from MRI data, to estimate the craniofacial midline. Of note, the focus of this study was the anterior cerebral falx plane, rather than the entire falx cerebri. We tested the null hypothesis that there was no difference in the repeatability, reproducibility, precision, and diagnostic outcomes of the mark point analysis method with that of the MRI-derived anterior cerebral falx plane method.

## Results

The intra-observer and inter-observer intra-class correlation coefficient (ICC) values for the 15 anthropometric indicators were all greater than 0.7. This indicated that the measurements made by either manual mark point analysis or MRI-based anterior cerebral falx plane were highly reproducible and repeatable (Table 1). A significant correlation of the ICC values (*P* < 0.01) was noted between the 2 methods. The anterior cerebral falx plane method showed a higher average coefficient of variation (0.029) than did the mark point analysis method (0.017), indicating slightly lower precision. However, the inter-group differences were not significant (*P* > 0.05).

**Table 1:** Coefficient of variation, intra-observer and inter-observer agreement for 3D cephalometric measurements.

	Coefficient of variation		Intra-observer ICC		Inter-observer ICC*	
	Method A	Method B	Method A	Method B	Method A	Method B
PosL	0.014 ± 0.013	0.009 ± 0.006	0.792	0.907	0.849	0.804
ZysL	0.013 ± 0.012	0.011 ± 0.009	0.737	0.867	0.715	0.907
EnLs	0.030 ± 0.026	0.016 ± 0.011	0.771	0.936	0.749	0.880
EnRs	0.015 ± 0.010	0.017 ± 0.009	0.908	0.919	0.805	0.905
ZysR	0.007 ± 0.006	0.007 ± 0.006	0.943	0.892	0.832	0.885
PosR	0.006 ± 0.005	0.007 ± 0.005	0.907	0.898	0.726	0.861
AlLs	0.016 ± 0.014	0.015 ± 0.016	0.890	0.896	0.712	0.816
AlRs	0.012 ± 0.012	0.020 ± 0.017	0.956	0.923	0.844	0.885
ChsL	0.011 ± 0.010	0.036 ± 0.028	0.954	0.733	0.733	0.889
ChsR	0.018 ± 0.014	0.027 ± 0.027	0.925	0.779	0.835	0.910
Me	0.045 ± 0.045	0.172 ± 0.186	0.981	0.875	0.957	0.870
PosR-GoRs-SP	0.031 ± 0.021	0.042 ± 0.028	0.947	0.897	0.863	0.932
GoRs-Me-SP	0.007 ± 0.006	0.010 ± 0.006	0.904	0.848	0.828	0.874
PosL-GoLs-SP	0.028 ± 0.017	0.035 ± 0.025	0.945	0.923	0.854	0.929
GoLs-Me-SP	0.008 ± 0.008	0.012 ± 0.012	0.879	0.744	0.793	0.798

Method A, mark point analysis of facial midline; Method B, anterior cerebral falx plane method.

*Inter-observer ICC was derived by using 2 time-point readings for 2 examiners.

**Figure 1:**
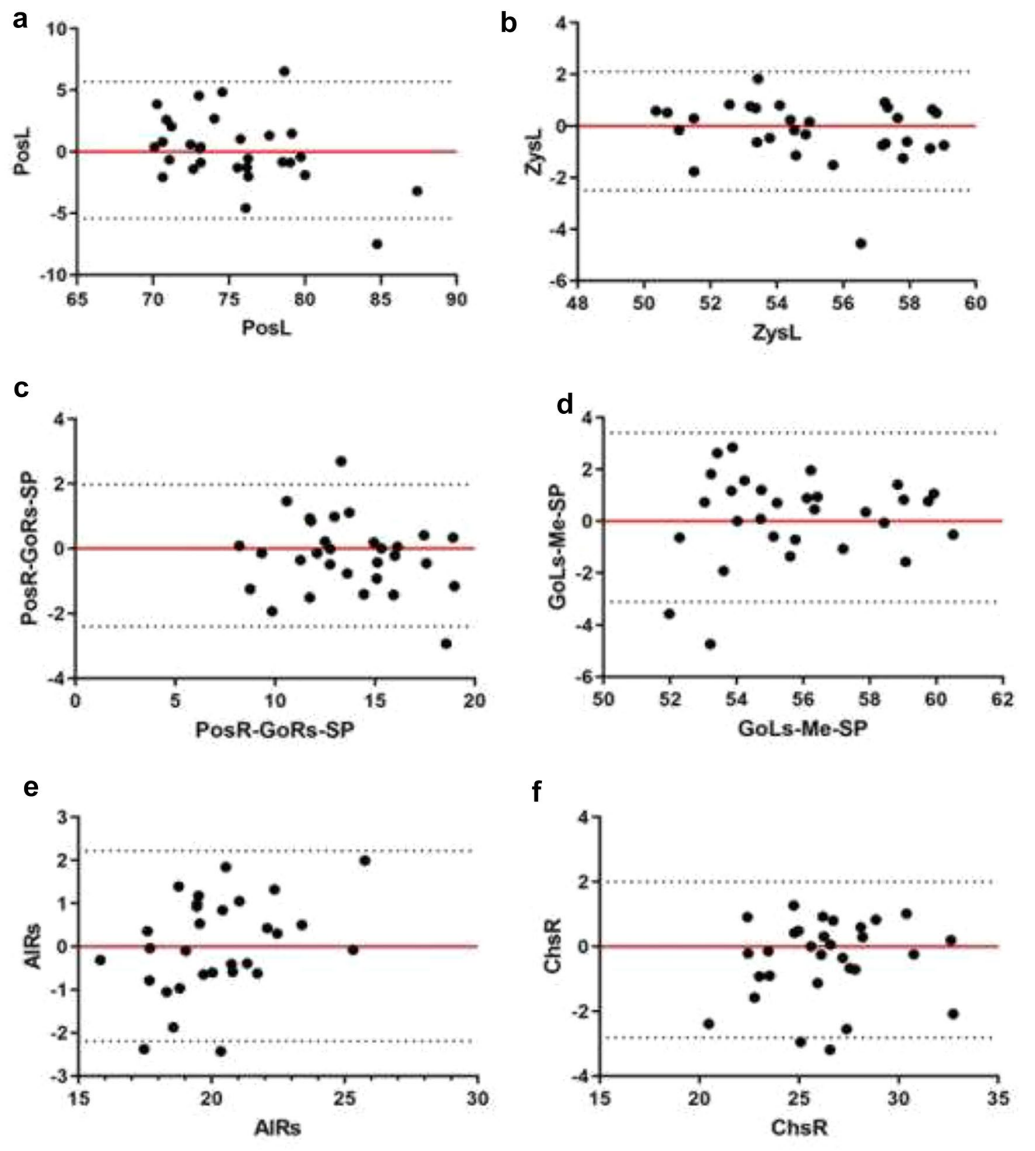
Bland–Altman plots show the differences between the measurements using the mark point analysis method and the cerebral falx plane method. Red lines represent the mean of all differences (bias), and black lines represent the 95% limits of agreement. The *x*- and y-axes represent the mean and standard deviation, respectively. Note the following exemplary measurements: (**a**) PosL-SP distance; (**b**) ZysL-SP angle; (**c**) PosR-GoRs-SP angle; (**d**) GoLs-Me-SP angle; (**e**) AlRs-SP distance; (**f**) ChsR-SP distance.

The intra-observer ICC for all the measurements when using the anterior cerebral falx plane was 0.869 ± 0.065 (range 0.733–0.936), and the ICC when using the mark point analysis method of craniofacial midline estimation was 0.8959 ± 0.0730 (range 0.737–0.981). The inter-observer ICC for all measurements using the anterior cerebral falx plane was 0.8763 ± 0.0417 (range 0.798–0.932), and the inter-observer ICC using the mark point analysis method was 0.8063 ± 0.0686 (range 0.712–0.957; Table 1). Figure 1 shows the Bland Altman plots, depicting the limits of agreement for the measurements using the 2 methods. The measured data obtained by the 2 methods were statistically comparable and no significant differences were noted for 2-group comparisons of all the measured metrics (*P* > 0.05; Table 2).

**Table 2:** Measurement data for the 2 craniofacial midline establishment methods.

	Method A	Method B	Mean difference	95% limits of agreement	P
PosL	75.32 ± 5.03	75.46 ± 3.85	0.14	− 5.430; 5.703	0.794
ZysL	55.17 ± 2.83	54.98 ± 2.59	− 0.19	− 2.492; 2.110	0.380
EnLs	19.82 ± 2.24	19.51 ± 2.20	− 0.31	− 2.391; 1.775	0.123
EnRs	19.40 ± 1.72	19.52 ± 1.95	0.12	− 1.460; 1.693	0.435
ZysR	54.61 ± 2.48	54.32 ± 2.31	− 0.30	− 2.509; 1.919	0.163
PosR	75.57 ± 2.67	75.15 ± 2.95	− 0.43	− 3.265; 2.413	0.118
AlLs	20.63 ± 1.90	20.57 ± 1.92	− 0.06	− 2.363; 2.237	0.772
AlRs	20.19 ± 2.10	20.20 ± 2.51	0.01	− 2.191; 2.215	0.955
ChsL	25.67 ± 2.54	26.08 ± 2.82	0.41	− 1.984; 2.804	0.076
ChsR	26.51 ± 2.92	26.11 ± 3.07	− 0.41	− 2.815; 2.005	0.082
Me	1.77 ± 0.88	1.69 ± 1.08	− 0.08	− 1.070; 0.908	0.387
PosR-GoRs-SP	13.82 ± 3.11	13.61 ± 2.92	− 0.21	− 2.396; 1.979	0.315
GoRs-Me-SP	53.43 ± 2.16	53.38 ± 2.15	− 0.05	− 2.197; 2.099	0.808
PosL-GoLs-SP	14.36 ± 3.01	14.54 ± 3.13	0.18	− 2.104; 2.469	0.399
GoLs-Me-SP	55.71 ± 2.48	55.87 ± 2.69	0.16	− 3.102; 3.412	0.613

Method A, mark point analysis of facial midline; Method B, anterior cerebral falx plane method.

## Discussion

This study investigated and confirmed that the MRI-based anterior cerebral falx plane is as repeatable and reproducible a method as manual mark point analysis for determining the craniofacial midline. These results support the application of MRI to replace other 3D sagittal positioning methods for craniofacial analysis^19–24^.

MRI does not rely on ionizing radiation and is non-invasive for radiological examination. It has been extensively employed for diagnosing jaw lesions, temporomandibular joint diseases, dental implants, orthodontic, and endodontic treatment^28^. Its safety and reliability for orthodontic diagnosis and treatment have been verified, while simultaneously alleviating the risks of radiation^29,30^. MRI has also been advocated for evaluating craniofacial asymmetry^26,31^. For orthodontic treatment, high-resolution MRI datasets can be converted into lateral cephalograms, which are highly consistent with conventional lateral cephalograms^13,32,33^. In recent years, numerous studies have confirmed the capability and reliability of 3-T MRI in 3D craniofacial measurement, and that it can replace CBCT examination as an effective instrument for cephalometry^33–35^.

Recently, several approaches for establishing the craniofacial median sagittal reference line have been proposed. The most widespread method uses 3 anatomical markers in the midline of the cranial face to construct the median sagittal plane^36,37^. The anatomical landmarks used frequently are the nasal root, butterfly saddle, skull base, and midpoint or posterior margin of the occipital aperture. Some studies have proposed the horizontal plane and the median sagittal plane^38,39^. These methods have limitations. For example, the facial hallmark point and external reference frame methods are subjective, under the natural head position^40^. The reference planes usually do not provide for anatomical variations and do not consider patients with cranial and maxillofacial deformities^41^. Jiang et al.^27^ reported using the anterior falx cerebri for evaluating 3D craniofacial features.

The results of the present study suggest that the midline of the anterior cerebral falx plane can be utilized as the midline for 3D craniofacial soft tissue analysis. Numerous studies^42^ have shown a high degree of consistency in the development of the forebrain and upper face as a modular developmental unit^43^, which are regulated by common signaling molecules. The occipital sickle was not used in the present study, since it has been reported to deviate from the midline and varies^44^. While the anterior cerebral falx plane is an internal cranial structure, it has been demonstrated via high resolution CT to stably represent the facial midline landmarks including nasion, anterior nasal spine, and mandibular symphysis^45^. The finding of our study is in accord with these studies, and the anterior falx cerebri, originating in the anterior pituitary fossa, can be used to establish the median sagittal plane. MRI without dedicated post-processing is applicable for clinical cephalometry^46^ and technical advances make low-cost portable MRI scanners^47^ a possibility. This suggests a sound basis for MRI-based anterior flax plane craniofacial midline assessment.

A natural head position is essential in performing craniofacial imaging analysis^48^. However, the present study design did not consider head position since it is not clear whether it affects establishment of a 3D coordinate system. In addition, the head-positioning device can deform the facial soft tissue during MRI acquisition, and muscle activity can also affect 3D craniofacial evaluation. There is also a need to evaluate the MRI-based anterior cerebral falx plane as a midline indicator in individuals with craniofacial asymmetry. These potential sources of variation should be addressed in future research.

Our experimental results suggest that the anterior falx plane is a valid and reliable craniofacial midline indicator. The visualization of soft tissue and black bone sequence-based assessment of bony structures may popularize MRI for orthodontic and craniofacial imaging^26,31^. However, considering that MRI is not widely applied clinically for 3D imaging in craniofacial assessment, unlike CBCT, the direct clinical application of these results is unlikely at this stage. Future studies with larger samples are needed to promote the application of MRI in digital orthodontics 3D craniofacial analysis.

In conclusion, the repeatability, reproducibility, precision, and diagnostic outcomes of MRI-based craniofacial midline estimation based on the anterior falx plane were comparable to that of conventional manual mark point analysis, indicating good agreement between the 2 methods. The utility MRI-based craniofacial midline estimation based on the anterior falx plane in future radiation-free 3D craniofacial analysis should be considered and verified further.

## Methods

### Study subjects

This was an observational, retrospective study. The protocol was approved by the Chifeng College Affiliated Hospital (No. fsyy202217), and all the procedures conformed with the Declaration of Helsinki. All participants provided written informed consent.

In September 2018, the data of 30 patients (14 males, 16 females, mean age 21 years, range 14–60 years) who had undergone MRI examination were collected retrospectively from the Radiology Department. For inclusion, each participant conformed to the following criteria: scanning was from the cranial roof to the lower edge of the mandible; the patient possessed complete dentition; and the teeth were in the median tooth position. A single experienced orthodontist and radiologist confirmed that there were no visible craniofacial asymmetry deformities evident on the MR images; there were no congenital developmental abnormalities such as cleft lip and palate; and no history of maxillofacial surgery or plastic surgery.

Sample size was estimated using PASS 21.0, with the parameters set at a confidence level of 90%, 6 observations per subject, confidence level width 0.2, no dropout, and an intraclass correlation of 0.73. This led to a computed sample size of 29, which was rounded off to 30.

### MR scanning and data processing methods

All images were obtained by the same technician using an 8NV head coil and a 1.5 Tesla System (GE Medical System, Signa HDxt; Table 3). Each patient was positioned conventionally for head imaging, with a median occlusal position of the upper and lower teeth.

**Table 3:** MRI scan parameters (T1, 3D-bravo) used for the soft tissue sequences.

Repetition time, ms	8.3
Echo time, ms	3.2
Flip angle, degree	13
Slice thickness, mm	1.2
Phase encoding	256
Frequency encoding	256
Zero filling interpolation	1024
Excitations, n	1
Echo train length, n	1

### Determination of the craniofacial midline on the MeVisLab workstation

#### Reference craniofacial midline established through conventional mark point analysis

**Figure 2:**
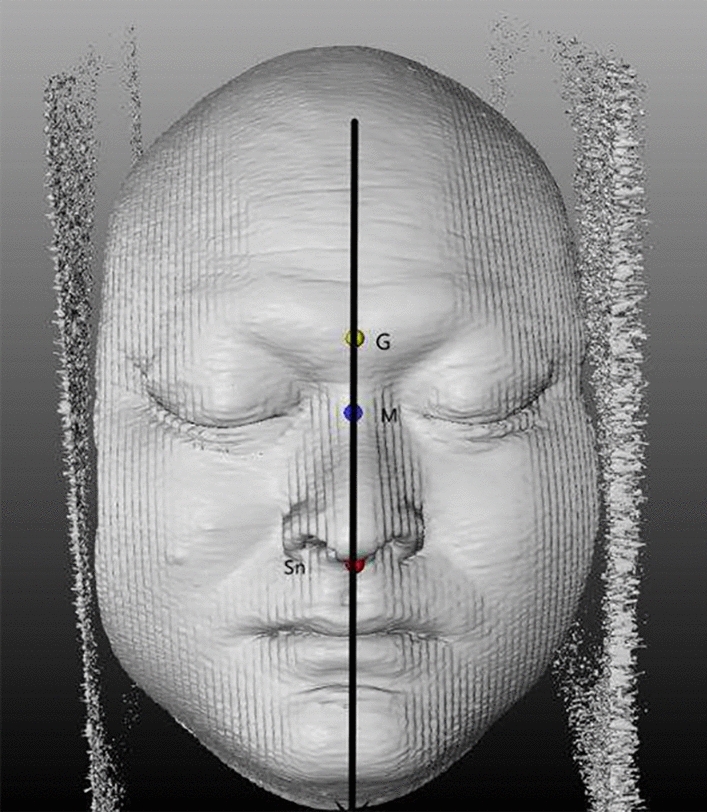
The soft tissue midline identification method. The black line represents the midline of the soft tissue surface. *G* glabella, *M* median midpoint of the inner canthus connection, *Sn* subnasale.

The mark point analysis method has been widely applied since the twentieth century^14,17,49^. The method uses, as the median, the sagittal line through the frontal point (G, glabella), subnasale (Sn), and the midpoint of the inner canthus connection (Fig. 2).

#### Craniofacial midline based on anterior cerebral falx plane in MRI

To perform the craniofacial midline estimation based on the anterior cerebral falx plane^27^, images were accessed using the multi-planar reconstruction function. The axial, sagittal, and coronal planes were input into the 3D coordinate system, selecting the yellow axis. The vertical axis represented a plane in 3D space.

**Figure 3:**
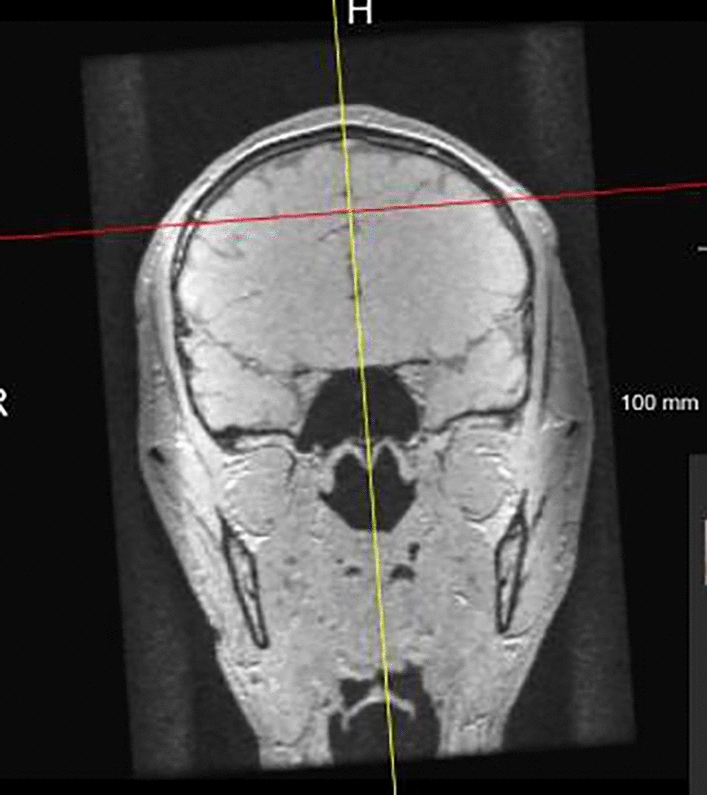
Coronal plane. The yellow line coincides perfectly with the anterior flax cerebri.

The following steps were performed to adjust the position of the vertical axis to coincide with the anterior cerebral falx (brain sickle) plane. Firstly, the position was adjusted to the coronal position in the pituitary socket so that the coordinate axis coincided with the anterior cerebral falx plane. This coinciding line was the intersection between the plane of the coordinate axis and the anterior cerebral falx plane at the coronal position, depicting the head in an upright position (Fig. 3).

**Figure 4:**
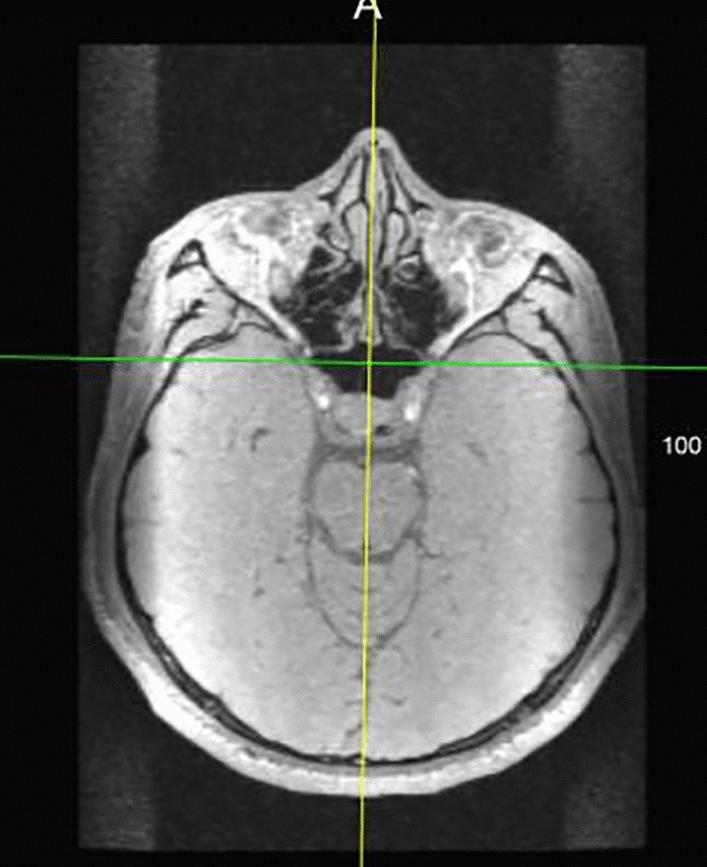
Axial plane. The yellow line coincides perfectly with the falx cerebri.

**Figure 5:**
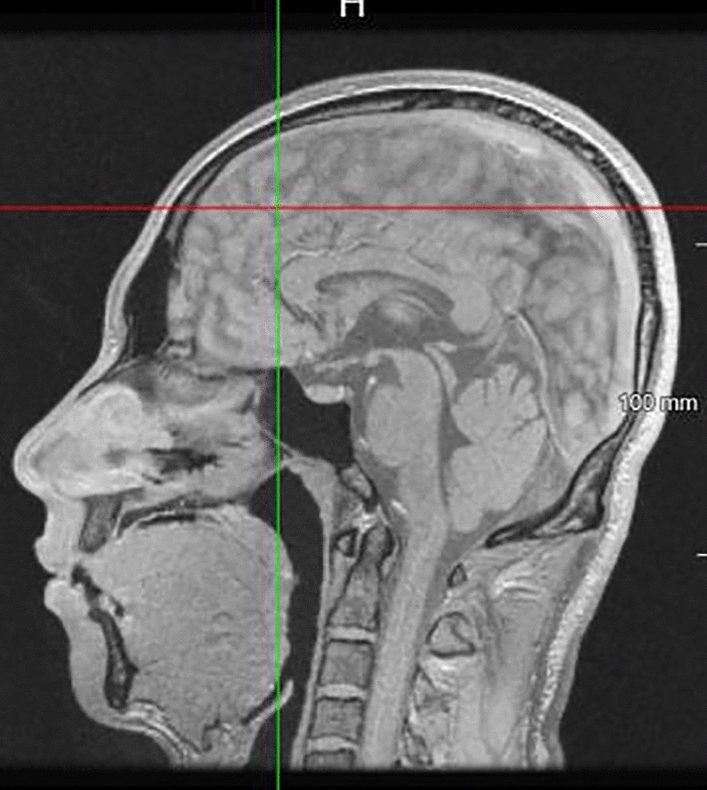
Anterior falx cerebri in the sagittal plane (anterior cerebral falx plane).

Keeping the yellow line steady, the angle of the yellow line to the anterior flax cerebri at the horizontal axis was observed: this was the plane's intersection angle represented by the yellow line and the anterior cerebral falx plane. The yellow line was rotated at the origin of the intersection so that it coincided with the falx cerebri, and was aligned with its plane. To maximize its overlap, the plane was then corrected on each horizontal axis in a craniocaudal direction (Fig. 4). Fine adjustment of the anterior cerebral falx plane was made in the sagittal position, which depicted a sickle membrane-like structure in the sagittal plane. Images of surrounding brain tissue, such as the sulcus gyrus, were avoided. The anterior cerebral falx images in the sagittal plane were highly reproducible (Fig. 5).

### Anthropometric landmarks and measurements

Selected well-established anthropometric metrics were measured based on landmark facial points and angles, including the distance to the established midline and the projection of the sagittal plane. The extensively adopted evaluation metrics in craniofacial analysis (cephalometric and anthropometric)^50^, are provided in Table 4. In the present study, the evaluation index point remained fixed, and the distance and angle of the planes were respectively measured.

**Table 4:** Anthropometric landmarks and measurements recorded.

Line distances to MSR sagittal midline	
PosL	Left tragus point (POS)
ZysL	Zygion of left zygomatic arch
EnLs	Left inner canthus point (ENS)
EnRs	Right inner canthus point (ENS)
ZysR	Zygion of right zygomatic arch
PosR	Right tragus point (POS)
AlLs	Left lateral alar margin (ALS)
AlRs	Right lateral alar margin (ALS)
ChsL	Left corner point (CH)
ChsR	Right corner point (CH)
Me	Soft tissue submental point
Angles	
PosR-GoRs-SP	Angle between the right mandibular branch line and the median sagittal line (right mandibular branch line: the line between the right tragus point POS and the right soft tissue mandibular angle GO point)
GoRs-Me-SP	Angle between the right mandibular body line and median sagittal line (right mandibular body line: the line between right soft tissue mandibular corner GO and submental point ME)
PosL-GoLs-SP	Angle between the left mandibular branch line and the median sagittal line (left mandibular branch line: the line between the left tragus point POS and the left soft tissue mandibular angle GO point)
GoLs-Me-SP	Angle between the right mandibular body line and median sagittal line (left mandibular body line: the line between left soft tissue mandibular corner GO and submental point ME)

*ALS* lateral alar margin, *CH* bilateral corner point, *ENS* inner canthus point, *GO* gonial point, *Me* soft tissue submental point, *POS* tragus point.

### Recording of measurements

All measurements were performed twice by 2 independently trained, experienced orthodontists, and each examiner repeated the measurements 3 weeks later. Each point was positioned 3 times, averaged, and recorded. The measurements were repeated under the same environmental conditions.

### Statistical methods

Statistical analyses were performed using SPSS (version 22) software. Descriptive data are summarized as mean ± standard deviation. The Shapiro–Wilk test was used to assess the normality of data distribution.

The agreement between the 2 methods was assessed by examining the correlation of the ICC values, Bland Altman plot analysis, and differences between the obtained craniofacial measurement data. Intra- and inter-observer agreements for the measurements for each method were determined by ICC values. Repeatability was represented by the intra-observer ICC, and reproducibility by the inter-observer ICC. The correlation between the ICC values obtained from the 2 methods was assessed by Spearman’s correlation. The precision of the measurements was estimated by computing the coefficient of variation for each measurement. Diagnostic outcomes were examined by testing the inter-group differences in the measurements obtained by the 2 methods. Between-group comparisons of measurement data were made using the independent samples *t*-test. The level of significance was set at *P* = 0.05 for all tests.

### Ethics approval and consent to participate

This was an observational, retrospective study. The protocol was approved by the Institutional Review Board and Medical Ethics Committee of Chifeng College Affiliated Hospital (No. fsyy202217) and all the procedures conformed with the Declaration of Helsinki. All participants provided written informed consent.

## Data Availability

The data that support the findings of this study are available from the corresponding author upon reasonable request.
